# Femoroacetabular impingement and classification of the cam deformity: the reference interval in normal hips

**DOI:** 10.3109/17453671003619011

**Published:** 2010-03-31

**Authors:** Thomas CB Pollard, Richard N Villar, Mark R Norton, E Darren Fern, Mark R Williams, David J Simpson, David W Murray, Andrew J Carr

**Affiliations:** ^1^Nuffield Department of Orthopaedics, Rheumatology and Musculoskeletal Sciences, NIHR Biomedical Research Unit, University of Oxford, and Nuffield Orthopaedic Centre NHS Trust, HeadingtonOxford; ^2^The Wellington Hospital, St John's WoodLondon; ^3^Royal Cornwall Hospitals NHS Trust, Treliske, TruroCornwall; ^4^Plymouth Hospitals NHS Trust, Plymouth, DevonUK

## Abstract

**Background and purpose:**

Most patients with femoroacetabular impingement (FAI) have a cam deformity, which may be quantified by measuring the alpha angle and anterior offset ratio (AOR). Knowledge of what constitutes a “normal” alpha angle and AOR is limited. We defined the reference intervals of these measurements from normal hips in the general population.

**Patients and methods:**

157 individuals from the general population were reviewed clinically and radiographically. 74 individuals with clinical evidence of hip disease or radiographic evidence of osteoarthritis (OA) were excluded, leaving a study group of 83 individuals (mean age 46 (22–69) years, 44 females) with normal hips. The alpha angles and AORs were measured from cross-table lateral radiographs taken in 15° internal rotation. A validation study consisting of a cadaver study and a measurement reliability study was also performed.

**Results:**

The mean alpha angle was 48° in men and 47° in women. The mean AOR was 0.19, the same in men and women. Thus, sexes were combined to derive 95% confidence intervals for the population mean alpha angle (46–49°) and AOR (0.18–0.20). The 95% reference interval for the alpha angle was 32–62° degrees, and for the AOR it was 0.14–0.24. The validation study confirmed that these measurements were resistant to a reasonable degree of variation in positioning and that the repeatability and reproducibility of the measurements was good.

**Interpretation:**

These reference intervals indicate that clinically and radiographically normal hips may have alpha angles and AORs that have previously been considered “abnormal”. The thresholds provided by this study will aid classification of individuals involved in longitudinal studies of FAI and OA, and may be of use to the practicing clinician in evaluating the young adult with hip pain.

## Introduction

Femoroacetabular impingement (FAI) may cause hip pain in young adults and damage to the labrum and chondral surfaces (Beck et al. 2005). While the main difference between a normal hip and a hip with FAI is abnormal joint morphology, the impact of this abnormal morphology on the joint is probably modulated by activity level and the durability of the chondro-labral junction. Surgical techniques to address the underlying morphological abnormalities have evolved with the concept of FAI. Hip arthroscopy (Bardakos et al. 2008), surgical dislocation (Beck et al. 2004), anterior arthrotomy (Clohisy and McClure 2005), and periacetabular osteotomy (Siebenrock et al. 2003) are now established techniques and improve symptoms in the short term. However, in addition to the absence of controlled trials of these interventions, our understanding of the etiology and natural history of FAI is limited. In order to answer these questions, classification of anatomy, activity, and biology is required to characterize individuals involved in cohort studies.

Most patients with FAI have a cam deformity, characterized by a reduction of offset or abnormal asphericity at the anterolateral femoral head-neck junction (Beck et al. 2005). This may be recognized by radiography (Clohisy et al. 2008), CT (Beaule et al. 2005), and MRI (Notzli et al. 2002), and quantified by measuring the alpha angle (Notzli et al. 2002) and anterior offset ratio (Eijer et al. 2001). Threshold values for the alpha angle have been suggested based on anteroposterior (AP) pelvic radiographs (Gosvig et al. 2007), but this view is not optimal for assessing the cam deformity (Goodman et al. 1997, Meyer et al. 2006). The cross-table lateral radiograph is recommended (Meyer et al. 2006) and is in routine clinical use (Clohisy et al. 2008), but there are no robust quantitative definitions of normal and abnormal anatomy based on this projection.

The aim of this study was to define reference intervals (Altman 1999) for the morphology of the proximal femur in the context of the cam deformity, based on cross-table lateral radiographs of asymptomatic individuals from the general population with a normal clinical examination and no osteoarthritis (OA). In order to validate the radiographic assessment, we measured the effect of variation in radiographic technique in a cadaver femora study and performed a measurement reliability study.

## Patients and methods

### Clinical study

157 individuals (mean age 48 (22–72) years, 79 females) were recruited prospectively. These were the spouses or partners of subjects involved in two cohort studies. The first study was a sibling study of FAI, and the second study involved a group of middle-aged adults considered to be at risk of OA (Spencer et al. 2005). For both of these studies, the spouses/partners were recruited as a control group; as they were not blood-relatives, they were considered to be representative of the general population. Both of these studies had ethical approval (OxREC B 07/H0605/145 April 2008, and 07/Q1605/26 May 2007). All subjects signed an informed consent form and underwent an interview and clinical examination by the first author. All were asked if they had ever had surgery on either hip, if they had been investigated for problems with either hip as a child, adolescent, or adult, and if they had experienced any pain or stiffness in either hip over the previous year. Activity level was recorded using the University of California at Los Angeles (UCLA) activity score (Amstutz et al. 1984). The recorded examination findings were: the presence of a positive Trendelenberg test, fixed flexion deformity, leg length discrepancy, groin tenderness, impingement sign (Klaue et al. 1991), and groin discomfort on passive movement. The range of internal rotation at 90 degrees of flexion was measured with a goniometer.

All participants underwent standardized AP pelvic and cross-table lateral radiography of each hip. The AP radiographs were performed with the patient supine and with the X-ray beam centered in the midline and on the point midway between the superior border of the pubic symphysis and a line drawn connecting the anterior superior iliac spines (Clohisy et al. 2008). A 15° wedge was placed underneath the femoral condyles of each leg to ensure that the recommended internal rotation of each hip was achieved (Clohisy et al. 2008). The tube-to-film distance was 120 cm. The cross-table lateral radiograph was taken with the index femur internally rotated 15° (Meyer et al. 2006), as standardized using the same 15° wedge placed underneath the femoral condyles. The beam was angled 45° to the hip, centered over the femoral head, with a tube-to-film distance of 120 cm (Clohisy et al. 2008). The presence of osteophytes in each hip on the AP radiograph was graded using an atlas of radiographic features in osteoarthritis (Altman and Gold 2007). The minimum joint space width was measured using the Picture Archiving and Communication System measuring tool (PACS, 2004; GE Healthcare UK Ltd., Little Chalfont, Bucks, UK).

**Figure 1.**
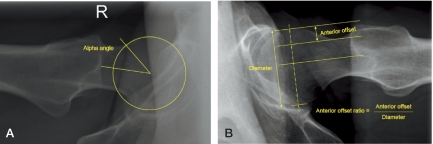
Method of measurement of the alpha angle (A) and anterior offset ratio (B).

The presence of a cam deformity was assessed on the lateral radiographs by measuring the alpha angle (Notzli et al. 2002, Clohisy et al. 2008) and anterior offset ratio (Eijer et al. 2001, Clohisy et al. 2008) (Figure 1). To calculate the alpha angle, a spherical template was placed over the femoral head and a line was drawn along the longitudinal axis of the femoral neck between the center of the femoral head and the center of the neck at its narrowest point. The point was then marked where the radius of curvature of the femoral head first exceeded that of the the template anteriorly. A straight line connecting this point and the center of the head was drawn. The angle between this line and the neck axis line is the alpha angle. The anterior offset ratio is calculated by first drawing a line along the central axis of the neck, which does not necessarily pass through the center of the femoral head. Parallel lines are then drawn along the anterior cortex of the neck and along the anterior outer part of the femoral head. The perpendicular distance between these latter two lines is the anterior offset. The anterior offset ratio (AOR) is calculated by dividing the anterior offset by the diameter of the femoral head (Eijer et al. 2001). Measurements were made by saving the digital images as TIFF files, which were analyzed in a custom-designed software program in Matlab (version R2007a; The Mathworks Inc., Natick, MA). The accuracy of this program had been tested and confirmed during its development.

### Exclusions and analysis

Because an individual's two hips cannot be considered to be independent of each other, we excluded any participant who had a single hip that was considered abnormal by the following criteria. Any patient who answered “yes” to having had surgery, or previous or current hip problems, or who had positive findings on examination (Trendelenberg, fixed flexion deformity, leg length discrepancy, groin tenderness, impingement sign, or irritability) was excluded. Following assessment of the AP radiographs for OA, we calculated sex-specific 95% reference intervals for the minimum joint space width. Any case with joint space narrowing below the lower threshold was excluded. All hips with any grade of osteophytosis were also excluded. Each hip was then assigned an overall grade for OA as described by Kellgren and Lawrence (1957), and any case with a hip with grade-I disease (doubtful osteoarthritis) or more was excluded. The remaining unexcluded cases were considered to have normal hips. The alpha angles and anterior offset ratios were calculated in the normal and excluded hips. In the normal group, the measurements for the left and right hip were averaged for each individual prior to calculation of the group mean and standard deviation.

### Validation study

#### Cadaver study.

Although the cross-table lateral radiograph taken with 15° of internal rotation of the femur is recommended for the radiographic assessment of the cam deformity (Meyer et al. 2006), it remains a 2-dimensional assessment that may be subject to variation due to variable patient positioning. To determine the influence of varying rotation on the variables of interest, 10 intact dry femora from the University Department of Anatomy were analyzed. 2 of these had a “pistol-grip” appearance suggestive of a cam deformity, with the other 8 having a normal appearance. Each femur was securely mounted on a wooden hinge with the posterior aspect of the condyles secured to the base. The hinge enabled a stepwise internal and external rotation relative to the horizontal, measured using an attached goniometer. Radiographs were taken centered on the femoral head, with a tube-to-femur distance of 100 cm and femur-to-film distance of 20 cm. Radiographs were recorded from the starting point of 15° external rotation through to 45° internal rotation, in 5° increments. These radiographs were also stored digitally and the same measurements recorded.

#### Reliability study.

All measurements were made by one observer (TCBP). Intraobserver repeatability of the alpha angle and anterior offset ratio was obtained by the re-measurement of 20 sets of radiographs (40 hips) from the clinical study after an interval of 4 weeks. Interobserver reproducibility was obtained by a second observer (MRW), using the same 20 sets of radiographs.

### Statistics

The one-sample Kolmogorov-Smirnov test was used to test whether the alpha angles and anterior offset ratios in the normal hips were normally distributed. The 95% reference intervals were calculated as the mean ± 1.96 SD (Altman 1999). Differences between groups and sexes were compared using Student's t-test. Chi-squared and Fisher's exact test were used to determine variations in the prevalence of hips within and outside the reference interval in the normal and excluded groups. Intra- and interobserver reproducibility of continuous variables was assessed by intraclass coefficients. All the statistical calculations were performed using SPSS statistical software version 13.0 and statistical significance was assumed when the p-value was < 0.05.

## Results

### Clinical study

35 females and 39 males were considered to have at least one abnormal hip and were therefore excluded (Table 1). 2 females had been treated nonoperatively for developmental dysplasia as infants, but they were also excluded on examination findings. None of the males had been investigated for childhood hip disorders. The 95% reference interval for the minimum joint space width was 2.9–5.1 mm in the females and 2.8–5.5 mm in the males. Of the 26 males who were excluded on the basis of radiographic OA alone, 23 were Kellgren and Lawrence (1957) grade I (osteophyte only) and the other 3 had joint space narrowing also (grade II). Of the females excluded on the basis of radiographic OA alone, only 1 had grade II disease and the remainder had grade I.

**Table 1.** Participants who were excluded

	Males	Females
No.	39	35
Mean age, years	54	46
Reason for exclusion		
clinical features only, no OA	7	14
OA only, no clinical features	26	16
clinical features and OA	6	5
OA: osteoarthritis.		

**Figure 2.**
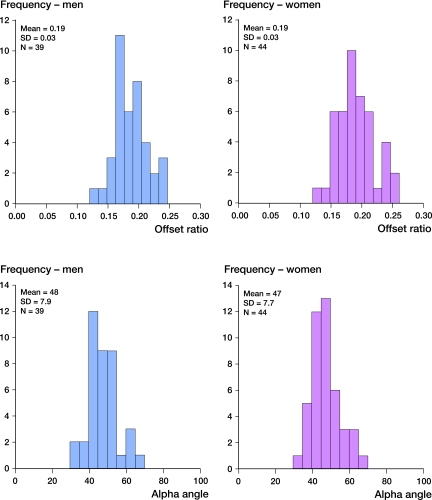
Frequency distribution of the anterior offset ratios and alpha angles in the participants included.

Thus, 83 participants (44 female and 39 male, mean ages 44.4 and 47.5 years, respectively (p = 0.2)) were considered to have “normal hips from the general population”. The mean UCLA activity scores in the normal and excluded participants were similar (7.9 and 7.6, respectively (p = 0.4)). Demographics, alpha angles, and anterior offset ratios of the normal participants are given in Table 2. Figure 2 shows the frequency distribution of the alpha angle and anterior offset ratios. The Kolmogorov-Smirnov test confirmed the Gaussian nature of these distributions. There was no difference in mean alpha angle and AOR between the sexes (p = 0.7 and p = 0.5 respectively). We therefore considered it reasonable to combine the data to derive confidence and reference intervals applicable to both sexes (Table 3). For the alpha angle, higher angles are clinically relevant and vice versa for the AOR. The prevalence of hips with either a high alpha angle, low AOR, or both, was statistically significantly higher in the excluded group than in the normal group (Table 4).

**Table 2.** Demographics, alpha angles, and anterior offset ratios for the participants who were included

	Males	SD	Females	SD
No.	39		44	
Mean age, years	48	12	44	11
age range	28–69		22–67	
Mean internal rotation				
at 90° flexion (°)	24	7	32	6
Mean alpha angle (°)	48	8	47	8
95% CI	45–50		45–49	
Mean AOR	0.19	0.03	0.19	0.03
95% CI	0.18–0.20		0.18–0.20	
AOR: anterior offset ratio.				

**Table 3.** Proposed 95% confidence and 95% reference intervals (mean ± 1.96 SD) of the alpha angle and anterior offset ratios, applicable to males and females

	Alpha angle (°)	Anterior offset ratio
Confidence interval	46–49	0.18–0.20
Reference interval	32–62	0.14–0.24

**Table 4.** Numbers of participants with at least one hip beyond the limits of the reference intervals (upper limit for alpha angle, lower limit for AOR) defined in Table 3. Percentages are given in parentheses

Number of cases with at least 1 hip with a	Females	Males	All	p-value (for all hips)
high alpha angle				
Normal	3 (7)	5 (13)	8 (10)	
Excluded	17 (44)	14 (36)	31 (42)	<0.001
low AOR				
Normal	1 (2)	3 (8)	4 (5)	
Excluded	4 (11)	9 (23)	13 (18)	0.02
high alpha angle AND low AOR				
Normal	0 (0)	0 (0)	0 (0)	
Excluded	4 (11)	5 (13)	9 (12)	0.001
AOR: anterior offset ratio.				

### Validation study

#### Cadaver study.

**Figure 3.**
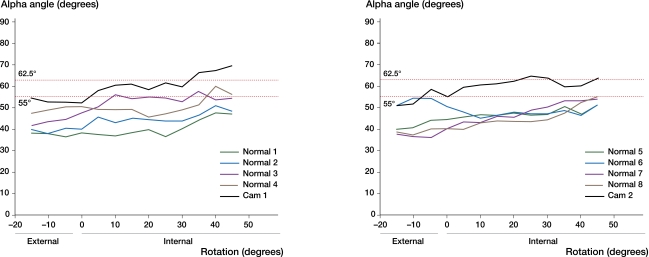
Graphs showing the relationship between rotation and alpha angle in the ten cadaver femora. The data are presented in 2 graphs to facilitate visual interpretation. There are four normal femora and one pistol-grip (cam) femur in each graph. The horizontal dotted lines represent 1 and 1.96 standard deviations above the mean.

**Figure 4.**
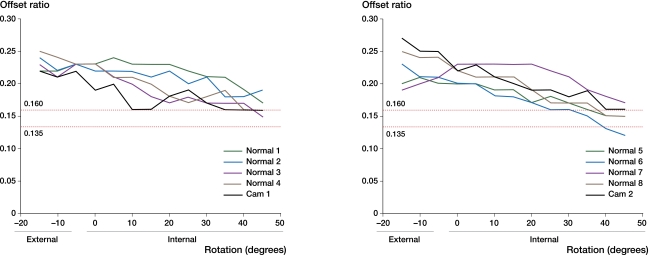
Graphs showing the relationship between rotation and anterior offset ratio in the ten cadaver femora. The data are presented in 2 graphs to facilitate visual interpretation. There are four normal femora and one pistol-grip (cam) femur in each graph. The horizontal dotted lines represent 1 and 1.96 standard deviations below the mean.

The alpha angle tended to increase, and the AOR to decrease, with progressive internal rotation. However, provided that the rotation error remained within an arc of 30° of the intended 15° internal rotation (i.e. neutral to 30° internal rotation), this effect was small (Figures 3 and 4).

#### Reliability study.

The interclass coefficient for the alpha angle was 0.84 (very good agreement) and for the AOR it was 0.73 (good agreement) (Petrie 2006). The intraclass coefficient for the alpha angle was 0.91 (very good agreement) and it was 0.75 for the AOR (good agreement).

## Discussion

In FAI, damage to the acetabular labral-chondral complex will depend on the degree of morphological abnormality of the joint, the biological durability of the cartilage and labrum, and an individual's activity level and type. Surgical intervention is based on the principles of restoration of normal anatomy and repair of the labral-chondral complex where possible. In the future, more complex adjuvant treatments will be introduced that may modify and enhance healing of the cartilage and labrum. In addition to interventional studies, longitudinal observational studies may clarify how the morphological abnormality in FAI develops and what the natural history is. In order to improve our understanding of the condition and its treatment, accurate classification of an individual's morphology, biology, and activity is necessary.

FAI occurs primarily as a result of an underlying bony abnormality, and therefore radiography is a valuable screening tool (Clohisy et al. 2008). Meyer et al. (2006) showed that the optimum projections to demonstrate asphericity of the femoral head were the Dunn view in 45° or 90° of flexion or the cross-table lateral in 15° internal rotation. The frog-leg lateral is also advocated (Clohisy et al. 2007). Measurement error may occur on account of variable positioning and observer reliability. Because the proximal femur has greater offset anteromedially than anterolaterally (Eijer et al. 2001, Meyer et al. 2006), the alpha angle increases and AOR decreases with progressive internal rotation. Our cadaver study demonstrated that provided the rotation of the femur was within a 30° arc, this variation was small. This is reassuring, as it is impossible to completely eliminate inconsistency of radiographic technique and image quality in clinical practice. Meyer et al. (2006) found the cross-table view to have the best observer reliability, and our repeatability and reproducibility compared favorably with other studies (Beaule et al. 2005, Clohisy et al. 2007, Neumann et al. 2009). Thus, we consider the measurement of the alpha angle and AOR from the cross-table lateral radiograph in 15° internal rotation to be a validated method for quantifying the morphology of the proximal femur in the context of the cam deformity.

The diagnosis of “normal” as opposed to “abnormal” based on continuous measurements is difficult, but is aided by the reference interval calculated from subjects representative of the healthy population (Altman 1999). It is important to appreciate that by definition, 5% of this healthy sample will have values beyond the reference interval. In clinical practice, the assessment of a patient presenting with symptoms due to FAI requires an evaluation of the femoral and acetabular anatomy, activity level, and status of the chondro-labral complex. In certain cases, the joint morphology may appear normal on screening radiographs and a more sensitive assessment using radial MRI may be necessary to detect subtle deformity (Rakhra et al. 2009). Thus, the information provided by reference intervals should not be used on its own to make a diagnosis (Altman 1999). Based on our data, patients presenting with symptomatic FAI and an alpha angle of 63° or more, or an AOR below 0.14 have proximal femoral anatomy that is beyond the reference interval. We would advocate a surgical strategy that addresses this deformity in such cases. Patients whose alpha angle and AOR are within the reference interval lie within a “gray” area. Given that there is no evidence to indicate the best surgical strategy in this situation, treatment should be determined on an individual case basis.

Although the alpha angle was originally described from MRI (Notzli et al. 2002), it is increasingly applied to plain radiography (Clohisy et al. 2008). Whether it is valid to apply these definitions across imaging modalities is questionable, but an alpha angle of greater than 50° has generally been considered “abnormal” (Notzli et al. 2002, Beaule et al. 2005). In practice, however, most patients with cam impingement have alpha angles in excess of 63° (Notzli et al. 2002, Beaule et al. 2005, Beaule et al. 2007, Clohisy et al. 2007) and so the reference intervals from our study would appear to be consistent. A few authors have suggested values for the “normal” alpha angle (Table 5), based on small cohorts using a variety of imaging modalities. The clinical assessment and radiographic assessment of OA in the subjects in these studies were variable, and whether they were truly representative of the healthy population is debatable; the imaging was frequently performed because the individuals had symptoms not attributed to the hip and they were therefore considered to be “normal”. Ours was a prospective study and all participants underwent a standardized clinical assessment by a single observer, and a standardized radiographic protocol from which the presence of OA in addition to assessment of proximal femoral morphology was made. The mean age, activity level, and racial background of our cohort were similar to those of patients undergoing surgery for FAI in our population (Beck et al. 2004). We therefore consider this sample to be representative of the population at risk of developing FAI. While a formal power calculation is not appropriate for this study design, as it is not comparative, Altman (1999) has provided guidance on the necessary sample size for reliable calculation of the reference interval. The number of observations should be greater than 50 and ideally over 200. As can be seen from Table 5, our study is the only one in the current literature with sufficient sample size to satisfy these recommendations. Recruitment of asymptomatic subjects for this kind of study is difficult, and our strict exclusion criteria limited the number of observations on which calculations were made. However, limiting the exclusions may have introduced bias to the results—resulting in a higher threshold of the alpha angle and lower threshhold for the AOR (Table 4).

**Table 5.** Suggested mean and upper limit of the reference interval values for the alpha angle in the current literature, from studies of asymptomatic individuals, with a lateral (or equivalent) radiological projection

Author	Imaging modality	No. of individuals (hips)	Alpha angle (°)			
			Mean	SD	Range	Upper limit 95% reference interval
Notzli et al. (2002)	MRI	35 (35)	42	2	33–48	50 a
Beaule et al. (2005)	CT	12 (20)	44	5	36–50	53
Clohisy et al. (2007)	Frog-lateral radiograph	24 (24)	44	12	30–92	67
Clohisy et al. (2007)	Cross-table lateral radiograph (neutral rotation)	24 (24)	47	15	31–76	77
Present study	Cross-table lateral radiograph (15° internal rotation)	83 (166)	47	8	30–70	62
**^a^** Suggested by authors						

For the AOR, the original description by Eijer et al. (2001) included 10 asymptomatic hips of unspecified gender, using cross-table radiographs in neutral rotation. The mean AOR was 0.21 with a standard deviation of 0.03 (0.14–0.25), suggesting a lower limit of the reference interval of 0.145. Beaule et al. (2007) proposed an AOR of less than or equal to 0.15 as being a risk for impingement, and Clohisy et al. (2009) found a mean AOR of 0.19 (0.10–0.28) in 22 control hips. These figures are in keeping with our data.

We found the mean alpha angle and mean AOR to be similar in normal males and females, which is interesting given that most patients with cam impingement are male (Beck et al. 2005). These results are in keeping with those of Beaule et al. (2005) and Ito et al. (2001), and suggest that the anatomy of the femoral head-neck junction in normal subjects is not inherently different between the sexes. Goodman et al. (1997) identified an overall prevalence of “post-slip” morphology of 8% in a study of 2,665 skeletons, defined by the degree of posterior tilt of the head relative to the neck axis together with a number of descriptive terms. A high proportion of the Goodman cohort were black Americans. To enable valid comparison with our Caucasian cohort, selection of the white males and females from the Goodman study reveals a rate of post-slip morphology of 7.4% and 4.9% respectively, and therefore 6.1% overall. In our study, 9 individuals (6%) had at least 1 hip with an alpha angle and AOR beyond the limits of the reference interval. The sex-specific rates were 6% and 5% in males and females, respectively. The alpha angle may be falsely elevated by secondary bony deposition, as a reactive response in FAI (Eijer et al. 2001), or by osteophyte formation as part of OA. The AOR may be less sensitive to these secondary changes. Considering these factors with the prevalence data above, we would recommend that the alpha angle and AOR be used in conjunction.

Our study has a number of limitations. We did not specifically assess the morphology of the acetabulum because our protocol did not allow standardization of pelvic tilt. We considered that subjects with substantially abnormal acetabular morphology would have been detected by the clinical assessment or development of OA. Neumann et al. (2009) recently studied a cohort of FAI patients who had undergone FAI surgery to restore an appropriate relationship of the acetabular rim and proximal femur, as quantified by “normal” internal rotation (20–25°) in flexion. From the postoperative radiographs, they proposed a “normal” alpha angle of 43°. The mean internal rotation at 90° of flexion in our cohort was similar (Table 2), supporting an appropriate relationship of the acetabular rim and proximal femur. MRI and CT are more sensitive than radiography (Dudda et al. 2009) because they provide morphological information in three dimensions, and also offer the opportunity to assess femoral neck version, which is of relevance to FAI and OA (Tonnis and Heinecke 1999, Ito et al. 2001) and which was not possible with our method. MRI has the added advantage of soft-tissue imaging, which may include the detection of non-osseous bumps at the head-neck junction. However, these modalities are more expensive, time consuming, and less readily available—which may limit their utility in large-scale trials. There is also potential for error in selecting which slice to take the measurement from (Rakhra et al. 2009), and even if the slice chosen is standardized, the assessment will remain prone to error on account of variable leg rotation. Our study group may have been subject to recruitment bias, those with symptoms being more likely to participate. However, this problem was eliminated by excluding such individuals from the reference interval calculation. In theory, partners of patients with FAI may themselves be more active and thus be at higher risk of developing FAI, but again the exclusion criteria eliminated this effect. This was a cross-sectional study. Some of the normal hips may become abnormal over time and it could be argued that one should study the elderly to gain ultimate data on normality. However, the strict clinical and radiographic exclusion criteria minimized the probability of inclusion of subjects who although currently normal are likely to become abnormal in the future.

In summary, the assessment of an individual presenting with FAI should account for the acetabular and femoral morphology, activity level, and damage to the chondro-labral complex. We have defined and validated 95% reference intervals based on cross-table lateral radiographs to describe the morphology of the proximal femur in the context of FAI. These will aid classification of individuals involved in observational and interventional longitudinal studies, and may be of use to the practicing clinician in assessing patients presenting with hip pain, and in planning their surgical strategy.
